# Reinfection with *Streptococcus suis* analysed by whole genome sequencing

**DOI:** 10.1111/zph.12528

**Published:** 2018-10-10

**Authors:** Niels Willemse, Arie van der Ende, Constance Schultsz

**Affiliations:** ^1^ Department of Medical Microbiology Academic Medical Center Amsterdam The Netherlands; ^2^ Department of Global Health Amsterdam Institute for Global Health and Development, Academic Medical Center Amsterdam The Netherlands; ^3^ Netherlands Reference Laboratory for Bacterial Meningitis Academic Medical Center Amsterdam the Netherlands

**Keywords:** bacterial meningitis, reinfection, *Streptococcus suis*, whole genome sequencing

## Abstract

A butcher with chronic dermatitis presented with a second episode of *Streptococcus suis* meningitis, 8 years after the first episode. To distinguish between reinfection and persistent carriage, we compared the two *S. suis* isolates using whole genome sequencing. We investigated whole genome sequences of the *S. suis* isolates by means of substitution rates and population structure of closely related strains in addition to available clinical information. Genome‐wide analyses revealed an inserted region consisting of 12 genes in the first isolate and the calculated substitution rate between the isolates suggested infections were caused by highly similar, but unrelated strains. Continuous occupational exposure likely resulted in reinfection with *S. suis* in a butcher.

**Impacts**

- We report the first genomic comparison between consecutive *Streptococcus suis* strains, isolated from a Dutch butcher with recurring meningitis.
- We conclude this reinfection was the result of similar yet unrelated strains based on genomic analyses.
- Professionals in continuous close contact with pigs should be vigilant of reinfection even after previous *S. suis* related illness as natural infection may not provide protection against future infections.

## 1 INTRODUCTION

*Streptococcus suis* is a Gram‐positive bacterium with zoonotic potential, causing meningitis, septicaemia and arthritis (Wertheim, Nghia, Taylor, & Schultsz, 2009). *Streptococcus suis* is rarely found in healthy individuals, but is a commensal of pigs and is carried in the upper respiratory and the gastrointestinal tracts of up to 100% of pigs in the Netherlands.

The *S. suis* serotype is determined by the antigenic properties of the polysaccharide capsule. Serotype 2 is responsible for infections in pigs and causes the majority of zoonotic infections (Goyette‐Desjardins, Auger, Xu, Segura, & Gottschalk, 2014). In the Netherlands, zoonotic infections with *S. suis* are observed predominantly in persons who have been in close contact with pigs, such as hunters, butchers and farmers (van de Beek, Spanjaard, & Gans, 2008). Despite passive surveillance through the Netherlands Reference Laboratory of Bacterial Meningitis (NRLBM), underreporting of *S. suis* meningitis cases still occurs (van de Beek et al., 2008; Wertheim et al., 2009). Vaccines that protect against *S. suis* infection are not available for human use.

We describe a middle‐aged male butcher who was known to suffer from chronic dermatitis and to carry porcine MRSA since 2007. On 19 April 2015, the patient presented with fever, headache, vomiting and diarrhoea. *Streptococcus suis* was cultured from cerebrospinal fluid (CSF). The isolate was submitted to the NRLBM, where it was linked to another isolate from CSF from the same patient isolated in 2007. Using whole genome sequencing, we investigated genetic differences between the 2007 isolate, 2071319, and the 2015 isolate, 2150651, potentially explaining this recurrent infection.

## 2 MATERIALS AND METHODS

The *S. suis* isolates were sequenced as described previously using paired‐end MiSeq sequencing (Willemse et al., 2016). We performed MLST using (https://pubmlst.org/ssuis) and annotated the genomes using Prokka 1.9 (https://github.com/tseemann/prokka). Roary (Page et al., 2015) was used to calculate core and pangenomes. For substitution rate calculation, SNPs were determined by mapping sequencing reads against the closely related reference genome of P1/7 using SMALT (https://sourceforge.net/projects/smalt), to include SNPs in intergenic regions, which are typically not included in a core genome, in the analysis. SNPs were extracted using Samtools (Li, 2011). Mappings were inspected using Artemis (https://www.sanger.ac.uk/science/tools/artemis). SNPs for phylogenetic analysis were extracted from the core genome alignment of Roary using SNP‐sites (https://github.com/sanger-pathogens/snp-sites). Maximum likelihood trees were generated with RAxML 8.1.6 (https://github.com/stamatak/standard-RAxML) and run until convergence at the bootstopping criterion.

## 3 RESULTS

Isolate 2071319 (ERS902349) was previously included in a genomic comparison of *S. suis* isolates from the Netherlands (Willemse et al., 2016) whilst isolate 2150651 (ERS1669548) was sequenced using identical methods. The 2071319 and 2150651 genomes were 2,048,581 and 2,036,490 nucleotides in length, respectively, and both belonged to ST1 and were serotype 2. GC contents were 41.17% and 41.23%, respectively. However, 2071319 contained 1988 coding sequences (CDS) whilst 2150651 contained 1970 CDS. The core genome of the two isolates comprised 1914 genes, leaving 31 genes in the accessory genome of which 24 genes belonged to 2071319 and seven genes to 2150651 (Table 1).

**Table 1:** List of accessory genes not shared between isolate 2071318 and 2150651 as determine by the Roary pangenome pipeline

Isolate	Draft genome gene location	Predicted protein function	Protein length	Nearest BLASTP reference protein
2071319	220	Mac family protein	1,084	WP_012775646.1
	380	Competence/damage‐inducible protein A	272	WP_012774894.1
	753	IS110 family transposase	242	CYX90486.1
	760	Minor spike protein H	202	EQJ03522.1
	891	Serine protease	712	WP_053866547.1
	937	Site‐specific integrase	436	WP_011922382.1
	938	hypothetical protein	76	WP_011922383.1
	939	Replication initiator protein	412	WP_014636592.1
	940	Hypothetical protein	174	WP_011922385.1
	941	Hypothetical protein	101	WP_012775097.1
	942	Hypothetical protein	433	WP_012775098.1
	943	Transcriptional regulator	68	WP_012775099.1
	944	Membrane/hypothetical protein	325	WP_011922387.1
	945	Hypothetical protein	279	WP_011922388.1
	946	Hypothetical protein	134	WP_012028114.1
	947	Hypothetical protein	340	WP_012775100.1
	948	Hypothetical protein	538	WP_011922392.1
	1,018	Hypothetical protein	108	WP_012775144.1
	1,052	Peptidase C26	67	CYV04131.1
	1,083	Penicillinase repressor (89% ID)	99	WP_011921706.1
	1,213	Cell surface protein (SadP)	902	WP_074392131.1
	1,223	ABC transporter ATP‐binding protein	275	WP_074411925.1
	1,362	*N*‐acetylmuramoyl‐l‐alanine amidase	373	WP_074415670.1
	1578	RNA helicase	467	WP_041179122.1
2150651	220	IgM protease (Mac family protein)	1,141	WP_011922092.1
	754	Minor spike protein	328	WP_000466547.1
	908	Hypothetical protein	100	WP_012775088.1
	1,067	Transcriptional regulator	156	WP_011921706.1
	1,197	Cell surface protein (SadP)	765	WP_012775427.1
	1,240	Oxidoreductase	66	WP_044764778.1
	1896	IS110 family transposase	276	WP_061843547.1

Part of the accessory genome of 2071319, spanning genes 937–948, encoded an integrase, replication initiator protein, transcriptional regulator, but mostly hypothetical proteins without predicted domains. The mean GC content of these contiguous genes was 30.6% and lower than the GC content of the whole genome. Among the remaining accessory genes is the *sadP* gene, encoding the Streptococcal Adhesion Protein (SadP), which had less than the 95% amino acid identity (84.8%) limit as set by Roary due to the presence of two fewer repeats. Mac family proteins also showed <95% identity due to different number of repeats, and the IS110 family transposases showed overlap, but had much lower protein identity. Other proteins did not show similarities.

Mapping of sequencing reads against the closely related reference genome of strain P1/7 yielded 74 SNPs and 24 indels in 2071319 and 100 SNPs and 15 indels in 2150651. There were 35 SNPs and seven indels shared between 2071319 and 2150651 against P1/7 resulting in 104 SNPs and 25 indels between 2071319 and 2150651. We did not identify regions of high SNP density, indicating these SNPs should be attributed to mutations instead of recombination. Using the SNPs, we estimated a required substitution rate of 6.36·10^−6^ substitutions per site per year (104 SNPs divided by the average genome size of 2071319 and 2150651, divided by 8 years), which is almost tenfold higher compared to the recently calculated substitution rate of 8.58 × 10^−7^ for related ST7 isolates in China (Du et al., 2017). It is also higher than the rates calculated for *S. pneumoniae* (1.57·10^−6^) and *S. aureus* (3.3·10^−6^) strains (Croucher et al., 2011).

**Figure 1:**
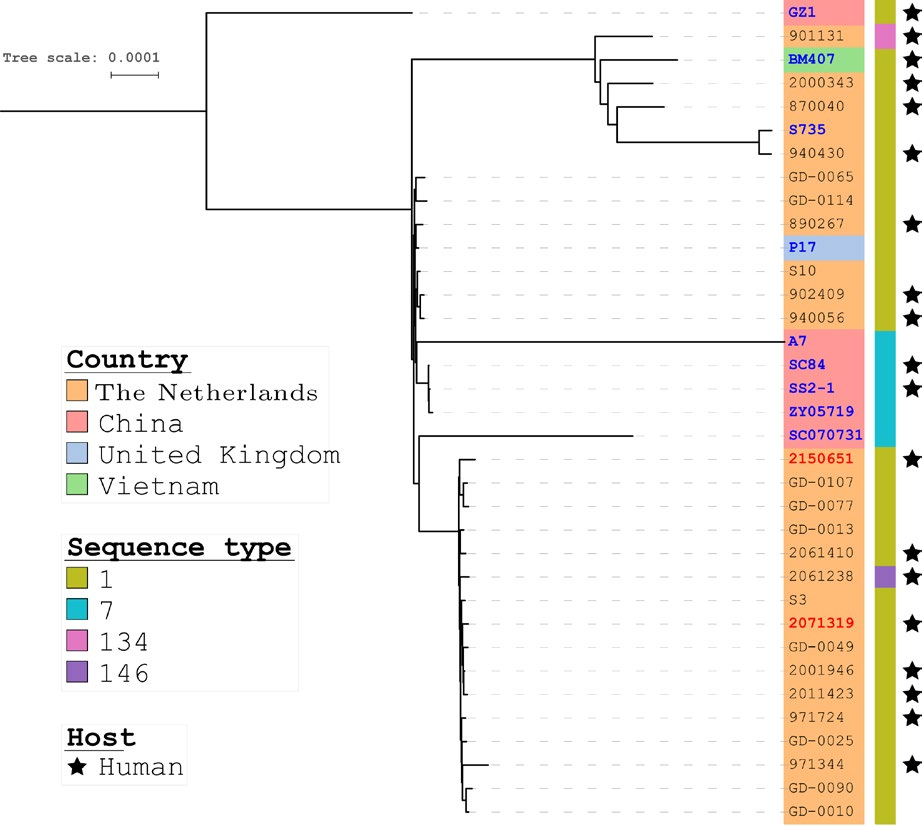
Unrooted maximum likelihood tree demonstrating the phylogenetic structure of core genomes of *Streptococcus suis* serotype 2 CC1 isolates from the Netherlands and international CC1 reference genomes. Country of isolation is indicated as well as the sequence type of each isolate. Black stars indicate *s. suis* isolates from human patients. Complete reference genomes are indicated in blue, and isolates 2071319 and 2150651, both responsible for infecting the same patient, are indicated in red. The tree was generated using SNPs in the core genome using RAxML until it converged at the bootstopping criterion, which was at 650 bootstraps [Colour figure can be viewed at wileyonlinelibrary.com]

We compared isolates 2071319 and 2150651 with all complete genomes of serotype 2 isolates, belonging to MLST clonal complex 1 (CC1), and included available draft genomes from the Netherlands (Figure 1, Supporting Information Table S1). Single Locus Variants (SLV) of ST1 were included in this analysis because single SNPs in the MLST housekeeping genes may not be representative of variation across the genome. Four main clusters could be observed. Outliers in this tree are GZ1 as well as A7 and SC070731 (both ST7 isolates) with long diverging branches (Supporting Information Figure S1). The cluster consisting of 16 isolates including the isolates 2071319 and 2150651 resulted in a core genome of 1886 genes with an accessory genome of 129 genes, which was slightly smaller than the shared core genome between isolates 2071319 and 2150651. A maximum likelihood tree was again generated to create the highest resolution among the closest related isolates (Supporting Information Figure S2). Whilst isolates 2071319 and 2150651 cluster on the same branch, they cluster amongst other CC1 isolates from the Netherlands suggesting these two isolates are not more related to each other than to the other isolates. Using hierarchical clustering with the presence–absence matrix of the pangenome in R, we compared the accessory genome content of these 16 isolates (Supporting Information Figure S3). Isolates 2071319 and 2150651 clustered among other CC1 isolates from the Netherlands, but two main clusters of the accessory genome were separated in the dendrogram due to the presence or absence of the previously mentioned insertion in 2071319, indicative of co‐evolution of two CC1 subclones circulating in the Netherlands.

## 4 DISCUSSION

Our results suggest that it is unlikely that 2150651 was a descendant from 2071319 and indicate reinfection by two unrelated isolates belonging to different ST1 subclones. The isolates differed by an inserted region, with genes which were likely inserted as a whole, but the origin of this inserted sequence is not well understood. The isolates also had different SadP genes, previously characterized as an adhesin as well as a factor H binding protein which may contribute to zoonotic potential (Ferrando et al., 2017) and is considered a putative virulence factor of *S. suis* (Kouki et al., 2011; Pian et al., 2012).

Current evidence suggests that carriage of *S. suis* by humans in general is very rare (Nghia et al, 2011). Whilst potential carriage of *S. suis* due to continuous professional exposure to pigs (Bonifait, Veillette, Letourneau, Grenier, & Duchaine, 2014), combined with skin lesions related to his chronic dermatitis, cannot be ruled out in this patient, the genomic analysis does not suggest that infections occurred due to long‐term carriage of a single strain as the estimated substitution rate would be too high.

Reinfection with encapsulated bacteria, such as *Neisseria meningitidis* and *Streptococcus pneumoniae*, has been associated with host complement and immunoglobulins deficiencies (Lewis & Ram, 2014). Studies in C3‐ and C5R‐deficient mice indicated an increased susceptibility to *S. suis* resulting in severe infection in an intranasal mouse model (Seitz et al., 2014). A case of recurrent *S. suis* infections was reported in a patient after splenectomy (Francois, Gissot, Ploy, & Vignon, 1998). Whilst the patient did not have a medical history suggesting immunodeficiency, he did not consent to additional investigations to confirm or reject *S. suis* carriage or immunodeficiency, after his recovery.

In conclusion, we identified a patient with a *S. suis* reinfection on the basis of whole genome sequence analysis.

## CONFLICT OF INTEREST

The authors declare no conflict of interest.
