# Supplementary material for: Reinfection with Streptococcus suis analysed by whole genome sequencing
Source: Zoonoses Public Health. 2018 Oct 10;66(1):179–83. doi: 10.1111/zph.12528 (PMC7379552; doi:10.1111/zph.12528)
Supplement: Supplementary file 1 [file ZPH-66-179-s001.doc]

# Supplementary material


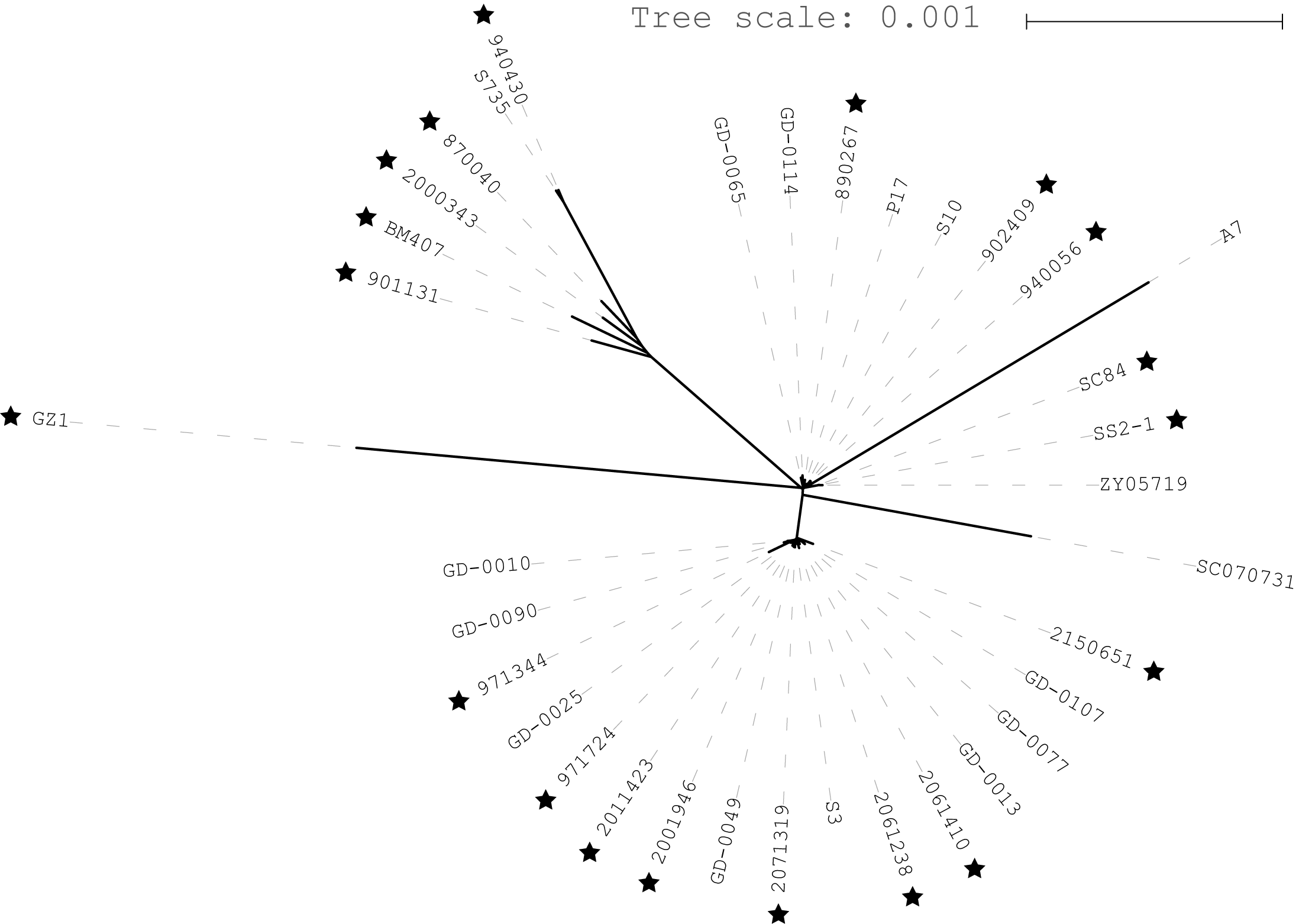


**Figure. S1**. An unrooted representation of the maximum likelihood tree based on the core genome alignment of 35 *S. suis* as depicted in **Figure 1**. Strains isolated from humans are indicated with black stars. RAxML was run on core genome alignment SNPs until it converged at the bootstopping criterion, which was at 650 bootstraps.


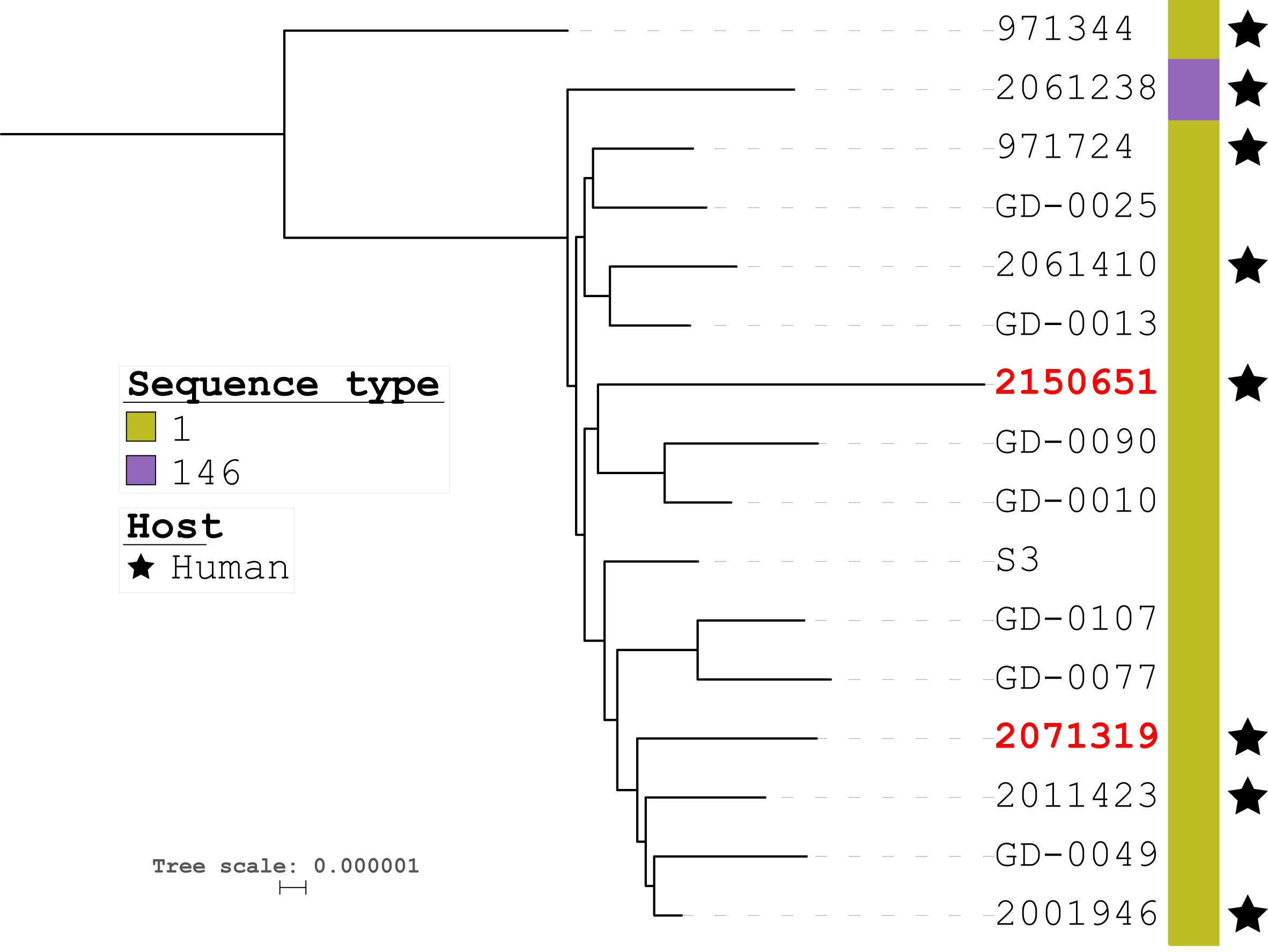


**Figure. S2**. A unrooted maximum likelihood tree based on the core genome alignment of 16 *S. suis* isolates from the Netherlands in the bottom branch of **Figure 1** containing both 2071319 and 2150651. By running Roary for these 16 isolates, we generated a slightly larger core genome of 1886 genes from which we extracted the SNPs, which provided higher resolution for the creation of a phylogenetic tree. The ST and the host of the isolates is indicated and isolates 2071319 and 2150651 are indicated in red. RAxML was run until it converged at the bootstopping criterion, which was at 1000 bootstraps.


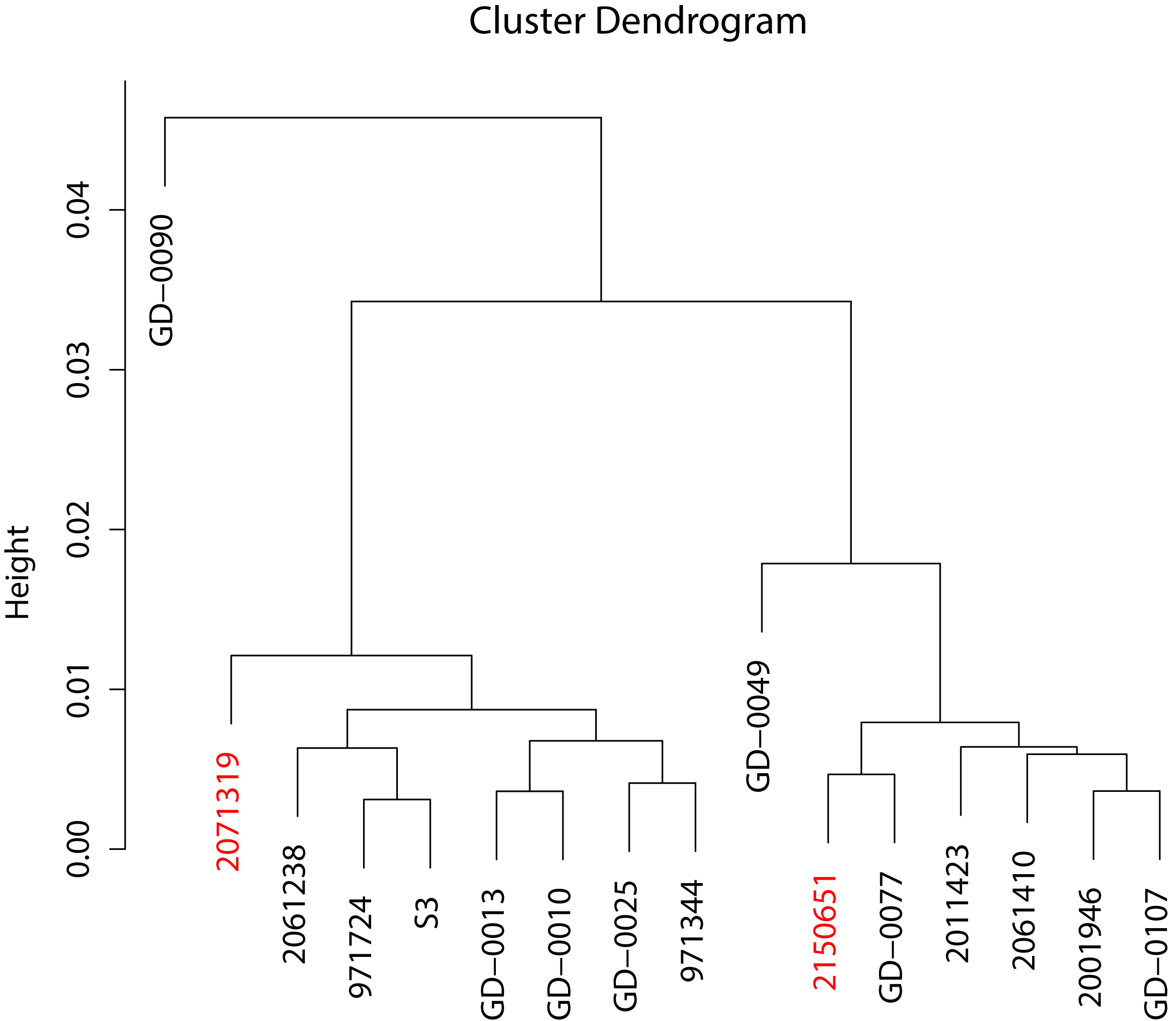


**Figure S3**. Dendrogram of the 129 genes in the accessory genome of the 16 *S. suis* isolates from the Netherlands in the bottom branch of **Figure 1**. A binary distance matrix was created and clustered hierarchically using hclust in R. Isolates 2071319 and 2150651 are indicated in red.

**Table S1**

Characteristics of included CC1 isolates from the Netherlands and complete CC1 genomes from the NCBI database as present on March 22^nd^, 2017.

| isolate | year | aroA | cpn60 | dpr | gki | mutS | recA | thrA | serotype | sequence  type | clonal complex | host | phenotype | source | accession | reads |
| --- | --- | --- | --- | --- | --- | --- | --- | --- | --- | --- | --- | --- | --- | --- | --- | --- |
| S735 | 1963 | 1 | 1 | 1 | 1 | 1 | 1 | 1 | 2 | 1 | 1 | Pig | Respiratory | Netherlands | CP003736 |  |
| S10 | 1982 | 1 | 1 | 1 | 1 | 1 | 1 | 1 | 2 | 1 | 1 | Pig | Meningitis | Netherlands | ERS902418 | ERR1055646 |
| 870040 | 1987 | 1 | 1 | 1 | 1 | 1 | 1 | 1 | 2 | 1 | 1 | Human | Meningitis | Netherlands | ERS902327 | ERR1055555 |
| S3 | 1988 | 1 | 1 | 1 | 1 | 1 | 1 | 1 | 2 | 1 | 1 | Pig | Meningitis | Netherlands | ERS902419 | ERR1055647 |
| 890267 | 1989 | 1 | 1 | 1 | 1 | 1 | 1 | 1 | 2 | 1 | 1 | Human | Meningitis | Netherlands | ERS902328 | ERR1055556 |
| 902409 | 1990 | 1 | 1 | 1 | 1 | 1 | 1 | 1 | 2 | 1 | 1 | Human | Meningitis | Netherlands | ERS902330 | ERR1055558 |
| 940056 | 1994 | 1 | 1 | 1 | 1 | 1 | 1 | 1 | 2 | 1 | 1 | Human | Meningitis | Netherlands | ERS902333 | ERR1055561 |
| 940430 | 1994 | 1 | 1 | 1 | 1 | 1 | 1 | 1 | 2 | 1 | 1 | Human | Meningitis | Netherlands | ERS902335 | ERR1055563 |
| 971344 | 1997 | 1 | 1 | 1 | 1 | 1 | 1 | 1 | 2 | 1 | 1 | Human | Meningitis | Netherlands | ERS902338 | ERR1055566 |
| 971724 | 1997 | 1 | 1 | 1 | 1 | 1 | 1 | 1 | 2 | 1 | 1 | Human | Meningitis | Netherlands | ERS902339 | ERR1055567 |
| GD-0010 | 1999 | 1 | 1 | 1 | 1 | 1 | 1 | 1 | 2 | 1 | 1 | Pig | Meningitis | Netherlands | ERS902364 | ERR1055592 |
| 2000343 | 2000 | 1 | 1 | 1 | 1 | 1 | 1 | 1 | 2 | 1 | 1 | Human | Meningitis | Netherlands | ERS902340 | ERR1055568 |
| 2001946 | 2000 | 1 | 1 | 1 | 1 | 1 | 1 | 1 | 2 | 1 | 1 | Human | Meningitis | Netherlands | ERS902342 | ERR1055570 |
| GD-0013 | 2000 | 1 | 1 | 1 | 1 | 1 | 1 | 1 | 2 | 1 | 1 | Pig | Meningitis | Netherlands | ERS902366 | ERR1055594 |
| 2011423 | 2001 | 1 | 1 | 1 | 1 | 1 | 1 | 1 | 2 | 1 | 1 | Human | Meningitis | Netherlands | ERS902343 | ERR1055571 |
| GD-0025 | 2001 | 1 | 1 | 1 | 1 | 1 | 1 | 1 | 2 | 1 | 1 | Pig | Meningitis | Netherlands | ERS902371 | ERR1055599 |
| GD-0049 | 2003 | 1 | 1 | 1 | 1 | 1 | 1 | 1 | 2 | 1 | 1 | Pig | Meningitis | Netherlands | ERS902378 | ERR1055606 |
| BM407 | 2004 | 1 | 1 | 1 | 1 | 1 | 1 | 1 | 2 | 1 | 1 | Human | Meningitis | Vietnam | FM252032 | NA |
| GD-0065 | 2004 | 1 | 1 | 1 | 1 | 1 | 1 | 1 | 2 | 1 | 1 | Pig | Meningitis | Netherlands | ERS902387 | ERR1055615 |
| GZ1 | 2005 | 1 | 1 | 1 | 1 | 1 | 1 | 1 | 2 | 1 | 1 | Human | Septicaemia | China | CP000837 | NA |
| GD-0077 | 2005 | 1 | 1 | 1 | 1 | 1 | 1 | 1 | 2 | 1 | 1 | Pig | Meningitis | Netherlands | ERS902393 | ERR1055621 |
| 2061410 | 2006 | 1 | 1 | 1 | 1 | 1 | 1 | 1 | 2 | 1 | 1 | Human | Meningitis | Netherlands | ERS902348 | ERR1055576 |
| GD-0090 | 2006 | 1 | 1 | 1 | 1 | 1 | 1 | 1 | 2 | 1 | 1 | Pig | Meningitis | Netherlands | ERS902400 | ERR1055628 |
| 2071319 | 2007 | 1 | 1 | 1 | 1 | 1 | 1 | 1 | 2 | 1 | 1 | Human | Meningitis | Netherlands | ERS902349 | ERR1055577 |
| GD-0107 | 2007 | 1 | 1 | 1 | 1 | 1 | 1 | 1 | 2 | 1 | 1 | Pig | Meningitis | Netherlands | ERS902408 | ERR1055636 |
| GD-0114 | 2008 | 1 | 1 | 1 | 1 | 1 | 1 | 1 | 2 | 1 | 1 | Pig | Meningitis | Netherlands | ERS902413 | ERR1055641 |
| 2150651 | 2015 | 1 | 1 | 1 | 1 | 1 | 1 | 1 | 2 | 1 | 1 | Human | Meningitis | Netherlands | ERS1669548 | ERR1934265 |
| P1/7 | NA | 1 | 1 | 1 | 1 | 1 | 1 | 1 | 2 | 1 | 1 | Pig | Meningitis | UK | AM946016 | NA |
| SC84 | 2005 | 1 | 1 | 1 | 1 | 1 | 1 | 3 | 2 | 7 | 1 | Human | Meningitis | China | FM252031 | NA |
| ZY05719 | 2005 | 1 | 1 | 1 | 1 | 1 | 1 | 3 | 2 | 7 | 1 | Pig | Diseased | China | CP007497 | NA |
| SC070731 | 2007 | 1 | 1 | 1 | 1 | 1 | 1 | 3 | 2 | 7 | 1 | Pig | Meningitis | China | CP003922 | NA |
| A7 | NA | 1 | 1 | 1 | 1 | 1 | 1 | 3 | 2 | 7 | 1 | Pig | Meningitis | China | CP002570 | NA |
| SS2-1 | NA | 1 | 1 | 1 | 1 | 1 | 1 | 3 | 2 | 7 | 1 | Human | Meningitis | China | CP018908 | NA |
| 901131 | 1990 | 1 | 61 | 1 | 1 | 1 | 1 | 1 | 2 | 134 | 1 | Human | Meningitis | Netherlands | ERS902329 | ERR1055557 |
| 2061238 | 2006 | 1 | 1 | 1 | 68 | 1 | 1 | 1 | 2 | 146 | 1 | Human | Meningitis | Netherlands | ERS902347 | ERR1055575 |
